# The impact of patient preference on dialysis modality and hemodialysis vascular access

**DOI:** 10.1186/1471-2369-15-38

**Published:** 2014-02-22

**Authors:** Patrick T Keating, Michael Walsh, Christine M Ribic, Kenneth Scott Brimble

**Affiliations:** 1 Division of Nephrology, Departments of Medicine and Clinical Epidemiology & Biostatistics, McMaster University, 50 Charlton Ave. E, Hamilton, ON L8N 4A6, Canada; 2 FRCP(C), Division of Nephrology, Department of Medicine, McMaster University, 50 Charlton Ave. E, Hamilton, ON L8N 4A6, Canada

**Keywords:** Peritoneal dialysis, Home hemodialysis, Central venous catheter, Predialysis education

## Abstract

**Background:**

Home-based dialysis, including peritoneal dialysis (PD) and home hemodialysis (HHD), is associated with improved health related quality of life and reduced health resource costs. It is uncertain to what extent initial preferences for dialysis modality influence the first dialysis therapy actually utilized. We examined the relationship between initial dialysis modality choice and first dialysis therapy used.

**Methods:**

Patients with chronic kidney disease (CKD) from a single centre who started dialysis after receiving modality education were included in this study. Multivariable logistic regression models were constructed to assess the independent association of patient characteristics and initial dialysis modality choice with actual dialysis therapy used and starting hemodialysis (HD) with a central venous catheter (CVC).

**Results:**

Of 299 eligible patients, 175 (58.5%) initially chose a home-based therapy and 102 (58.3%) of these patients’ first actual dialysis was a home-based therapy. Of the 89 patients that initially chose facility-based HD, 84 (94.4%) first actual dialysis was facility-based HD. The adjusted odds ratio (OR) for first actual dialysis as a home-based therapy was 29.0 for patients intending to perform PD (95% confidence interval [CI] 10.7-78.8; p < 0.001) and 12.4 for patients intending to perform HHD (95% CI 3.29-46.6; p < 0.001). Amongst patients whose first actual dialysis was HD, an initial choice of PD or not choosing a modality was associated with an increased risk of starting dialysis with a CVC (adjusted OR 3.73, 95% CI 1.51-9.21; p = 0.004 and 4.58, 95% CI 1.53-13.7; p = 0.007, respectively).

**Conclusions:**

Although initially choosing a home-based therapy substantially increases the probability of the first actual dialysis being home-based, many patients who initially prefer a home-based therapy start with facility-based HD. Programs that continually re-evaluate patient preferences and reinforce the values of home based therapies that led to the initial preference may improve home-based therapy uptake and improve preparedness for starting HD.

## Background

Home-based renal dialysis therapies for end-stage renal disease (ESRD), such as peritoneal dialysis (PD), home hemodialysis (HHD), or pre-emptive transplantation, are associated with improved health related quality of life [1,2] and reduced health resource costs [3]. It is widely accepted that patients with advanced stages of chronic kidney disease (CKD) should receive pre-ESRD modality education to ensure that patients able to initiate a home-based therapy have every opportunity to do so [4].

Despite the potential advantages of increased utilization of home-based dialysis therapies, its uptake remains low in North America [5]. The reasons for this vary, and include late referral of patients to nephrologists, insufficient education, lack of social supports for home therapies, and patient comorbidities [6-11]. Others have demonstrated that greater time spent on modality education is an independent predictor for initiating PD as the chronic modality [9]. Observational studies also suggest that the use of multi-disciplinary pre-dialysis clinics is associated with improved survival [12-14]. For these reasons, guidelines recommend timely referral of patients to a nephrologist to allow sufficient time for pre-ESRD modality education and dialysis access planning [15].

Studies indicate that when offered a choice, about half of patients choose a home-based therapy such as PD over facility-based hemodialysis (HD) [16,17]. However, estimates suggest only 7%-20% of incident ESRD patients actually utilize a home-based dialysis therapy [18-20]. Factors associated with choosing one modality but ultimately starting on a different modality are not well understood. To inform this issue, we studied characteristics associated with initiating a home-based therapy amongst patients with CKD that received pre-ESRD modality education.

## Methods

We performed a single centre, retrospective cohort study of patient enrolled in a multi-disciplinary clinic for CKD. This study was approved by the St. Joseph’s Healthcare Research Ethics Board.

### Study population

The Kidney Function Program at St. Joseph’s Healthcare Hamilton, Ontario, Canada is a multi-disciplinary pre-dialysis care program that serves an estimated population of 1,000,000 and began in January 2004. Patients were generally enrolled in this clinic when their serum creatinine reached 250 μmol/L and no further diagnostic investigations were necessary. Patients attending the clinic between its inception date and December 2012 who had initiated dialysis for a minimum of 30 days were included if they attended the clinic for at least 120 days, received pre-ESRD modality education, and declared an intended dialysis modality (or explicitly deferred the decision). Patients were excluded if they initiated dialysis on or prior to their modality education date or intended pre-emptive transplantation.

### Modality education

The clinic staff included a nephrologist, a nurse specialized in CKD management, a dietician, a diabetes nurse, and a social worker. All patients included in the study received formal modality education from one of two trained nurses. The education session lasted 120 minutes and covered materials on PD, HD, HHD including nocturnal HD, transplantation and conservative care (i.e., plan to not initiate dialysis in the event of ESRD). Separate group teaching sessions were also made available to supplement the one-on-one education. Family members were encouraged to attend and home-based therapies (and pre-emptive transplantation) were promoted although the ultimate decision was left to the patient.

### Data collection

Demographic [age, gender, diabetes mellitus (DM) status, presence of coronary artery disease (CAD), stroke, congestive heart failure (CHF), weight, and ethnicity], vascular access at HD initiation (central venous catheter [CVC], arteriovenous fistula [AVF] or arteriovenous graft) data and laboratory results (serum creatinine, MDRD eGFR, and albumin) were prospectively collected into a local clinical database. The dates for entry into the clinic, receipt of pre-ESRD modality education, decision regarding intended RRT modality, and actual RRT initiation date were prospectively recorded. Reasons why PD was not chosen initially as the intended RRT a (patient or physician decision) were also recorded. Specific reasons why patients did not initiate PD when they originally intended to were determined retrospectively from chart review. Distances from patient’s residence at the time of initiating RRT to the nearest HD unit were determined using Google Maps©.

### Outcomes

We evaluated the association between patient characteristics, including intended dialysis modality following patient education and actual initiated dialysis therapy. Initial dialysis therapy was defined as the treatment modality at 3-months after initiation of first ESRD therapy. Patients who had a PD catheter inserted prior to initiating any other modality were defined as having initiated on PD regardless of actual dialysis therapy initiated. Finally, in patients that initiated HD, we investigated the association between starting HD with a CVC and patient characteristics including their intended dialysis therapy.

### Statistical analysis

Descriptive statistics are presented as mean (standard deviation [SD]) or median (25th to 75th percentile) as appropriate. Continuous and categorical variables were compared between groups using either the one-way ANOVA or the Pearson chi-square test, respectively for variables normally distributed and the Kruskal-Wallis test or Mann–Whitney *U* test for non-normally distributed variables. Multivariable logistic regression models were constructed to assess the independent association of each variable with the outcomes described above. Independent variables were selected based on clinical importance and plausible association with the ability or desire to perform a dialysis modality. These variables included: intended therapy (HD, PD, HHD, undecided/conservative), sex, age, DM, CHF, eGFR, rate of eGFR decline in the CKD clinic, time from modality decision to initiation of dialysis, and distance to the nearest HD unit). To assess the predictors of starting HD with a CVC, we restricted the sample to patients that initiated HD and constructed a multivariable logistic regression model in which starting with a CVC was the outcome and the independent variables were the same as the previous model. All potential predictors were retained in all analyses. P-values of *<*0.05 for two-sided tests were considered statistically significant. All statistical analyses were performed using IBM SPSS Statistics Version 21.

## Results

**Figure 1:**
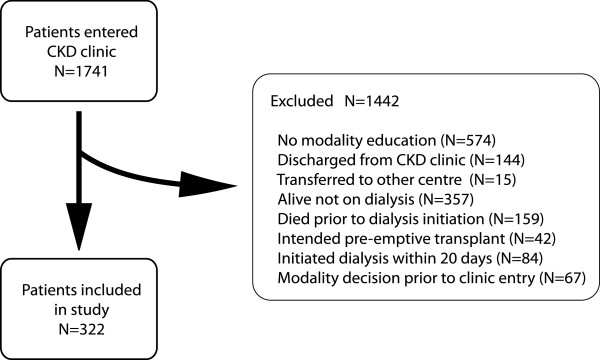
Study population selection.

Of 1741 patients who attended the CKD clinic, 299 patients who started dialysis after receiving modality education met study criteria. Reasons for exclusion are shown in Figure 1. Patients who intended to perform a home-based therapy tended to be younger with less CHF compared to those intending facility-based HD (Table 1). Patients who were undecided or intended conservative therapy were less likely to be Caucasian and started dialysis with a lower level of renal function compared to those who intended some form of dialysis. Compared to patients that initiated home-based dialysis, patients who initiated facility-based HD were older, and more commonly had CAD, CHF, and lower albumin (Table 2). Patients who initiated on HHD took longer to make a decision regarding their intended dialysis modality than those intending to perform facility-based HD or PD.

**Table 1:** Patient characteristics based on intended renal replacement modality

**Variable**	**HD (89)**	**HHD (21)**	**PD (154)**	**Conservative/undecided (35)**	**Total (299)**	**P value**
Age, years (SD)	72.1 (11.2)	63.1 (10.1)	68.2 (12.9)	69.5 (12.9)	69.1 (12.4)	0.012
Males, n (%)	59 (66.3)	15 (71.4)	82 (53.2)	22 (62.9)	178 (59.5)	0.13
Diabetes mellitus, n (%)	59 (66.3)	11 (52.4)	95 (61.7)	20 (57.1)	185 (61.9)	0.60
Coronary artery disease, n (%)	40 (45.5)	8 (38.1)	55 (36.9)	12 (35.3)	115 (39.4)	0.58
Previous stroke, n (%)	20 (23.0)	1 (4.8)	24 (15.8)	8 (22.9)	53 (18.0)	0.17
Congestive heart failure, n (%)	30 (34.5)	3 (14.3)	28 (18.4)	11 (31.4)	72 (24.4)	0.02
Weight, kg (SD)	85.7 (19.2)	98.2 (31.7)	84.7 (19.2)	84.4 (20.7)	85.9 (21.0)	0.048
Ethnicity, n (%)						
Caucasian	80 (89.9)	19 (90.5)	133 (86.4)	22 (62.9)	254 (84.9)	
Afro-Canadian	4 (4.5)	0 (0)	8 (5.2)	5 (14.3)	17 (5.7)	0.007
Aboriginal	2 (2.2)	2 (9.5)	2 (1.3)	3 (8.6)	9 (3.0)	
Other	3 (3.4)	0 (0)	11 (7.1)	5 (14.3)	19 (6.4)	
Creatinine, μmol/L (IQR)^a^	512.0 (405.0, 633.0)	535.0 (441.0, 662.5)	459.0 (418.0, 562.0)	622.0 (489.0, 783.0)	500.0 (418.0, 629.0)	0.001
eGFR, ml/min/1.73 m^2^ (SD)	9.4 (3.4)	10.1 (5.2)	9.6 (3.0)	7.7 (3.6)	9.4 (3.4)	0.022
Albumin, g/L (SD)	35.2 (5.3)	36.1 (6.4)	36.4 (4.5)	34.4 (4.0)	35.9 (5.0)	0.092
eGFR decrease,						
ml/min/1.73 m^2^/year (IQR)^a,b^	3.4 (1.6, 5.8)	3.6 (1.9, 7.5)	3.5 (2.2, 5.3)	4.0 (2.0, 7.3)	3.5 (1.9, 5.6)	0.83
Time- entry to decision, days (IQR)^a^	162.0 (85.0, 411.5)	128.0 (50.5, 369.5)	109.0 (40.5, 223.0)	157.0 (48.0, 379.0)	127.0 (49.0, 273.0)	0.010
Time-decision to initiation, days (IQR)^a^	334.0 (137.5, 618.0)	322.0 (145.5, 855.0)	325.5 (148.8, 325.5)	241.0 (66.0, 797.0)	323.0 (141.0, 731.0)	0.62
Distance to nearest HD unit, km (IQR)^a^	4.6 (2.2, 8.5)	5.5 (3.6, 10.7)	5.9 (3.0, 10.3)	6.4 (2.4, 10.9)	5.5 (2.6, 9.4)	0.22

Note: Means and standard deviations shown for normally distributed variables. Median and interquartile range values indicated by (^a^) for non-normally distributed variables. ^b^change in eGFR multiplied by (-1) based on available values preceding dialysis initiation.

*Abbreviations*: *HD* hemodialysis, *HHD* home hemodialysis, *PD* peritoneal dialysis, *kg* kilograms, *eGFR* estimated glomerular filtration rate based on 4-variable MDRD equation, *km* kilometers.

**Table 2:** Patient characteristics based on first renal replacement modality initiated

**Variable**	**HD (190)**	**HHD (14)**	**PD (95)**	**Total (299)**	**P value**
Age, years (SD)	70.3 (11.6)	61.9 (10.7)	67.9 (13.8)	69.1 (12.4)	0.024
Males, n (%)	119 (62.6)	11 (78.6)	48 (50.5)	178 (59.5)	0.048
Diabetes mellitus, n (%)	120 (63.2)	9 (64.3)	56 (58.9)	185 (61.9)	0.77
Coronary artery disease, n (%)	83 (45.4)	3 (21.4)	29 (30.5)	115 (39.4)	0.021
Previous stroke, n (%)	39 (21.0)	0 (0)	14 (14.7)	53 (18.0)	0.087
Congestive heart failure, n (%)	61 (32.8)	0 (0)	11 (11.6)	72 (24.4)	<0.001
Weight, kg (SD)	87.1 (21.1)	97.5 (30.6)	82.0 (18.1)	85.9 (21.0)	0.017
Ethnicity, n (%)					
Caucasian	161 (84.7)	12 (85.7)	81 (85.3)	254 (84.9)	
Afro-Canadian	13 (6.8)	0 (0)	4 (4.2)	17 (5.7)	0.72
Aboriginal	6 (3.2)	1 (7.1)	2 (2.1)	9 (3.0)	
Other	10 (5.3)	1 (7.1)	8 (8.4)	19 (6.4)	
Creatinine, μmol/L (IQR)^a^	524.5 (431.8, 524.5)	624.5 (486.0, 733.5)	446.0 (398.0, 532.0)	500.0 (418.0, 629.0)	<0.001
eGFR, ml/min/1.73 m^2^ (SD)	9.2 (3.6)	8.3 (3.2)	9.9 (3.0)	9.4 (3.4)	0.10
Albumin, g/L (SD)	35.2 (4.9)	37.5 (6.2)	36.8 (4.5)	35.9 (5.0)	0.013
eGFR decrease,					
ml/min/1.73 m^2^/year (IQR)^a,b^	3.2 (1.7, 5.5)	5.1 (2.7, 7.4)	3.7 (2.3, 5.4)	3.5 (1.9, 5.6)	0.056
Time- Entry to decision, days (IQR)^a^	147.5 (65.0, 359.3)	241.0 (42.5, 449.0)	108.0 (39.0, 190.0)	127.0 (49.0, 273.0)	0.011
Time-Decision to initiation, days (IQR)^a^	391.0 (139.0, 797.3)	436.0 (135.3, 589.0)	265.0 (146.0, 519.0)	323.0 (141.0, 731.0)	0.38
Distance to nearest HD unit, km (IQR)^a^	5.2 (2.5, 9.0)	5.6 (3.8, 16.5)	6.1 (3.0, 12.6)	5.5 (2.6, 9.4)	0.35

Note: Means and standard deviations shown for normally distributed variables. Median and interquartile range values indicated by (^a^) for non-normally distributed variables. ^b^change in eGFR multiplied by (-1) based on available values preceding dialysis initiation.

*Abbreviations*: *HD* hemodialysis, *HHD* home hemodialysis, *PD* peritoneal dialysis, *kg* kilograms, *eGFR* estimated glomerular filtration rate based on 4-variable MDRD equation, *km* kilometers.

One hundred seventy-five patients (54.3%, 154 PD and 21 HHD) intended to initiate a home-based therapy (Table 3). Ninety-one of the 154 patients (59.1%) who intended to perform PD actually did so; similar proportions were seen for those intending to initiate HHD. Overall, 102 of 175 (58.3%) participants planning to initiate home-based dialysis actually did so. This is in contrast to 84 of 89 (94.4%) patients intending to initiate facility-based HD and who went on to do so. Similarly, 33 of 35 (94.3%) patients that intended conservative therapy or deferred a decision went on to initiate facility-based HD.

**Table 3:** Distribution of patient numbers based on intended and actual ESRD therapies initiated

		**Actual modality**				**% who initiated intended modality**
		**PD**	**HHD**	**HD**	**Total**	
**Intended modality**	**PD**	91	2	61	154	59.1
	**HHD**	0	9	12	21	42.9
	**HD**	3	2	84	89	94.4
	**Undecided**	1	0	14	15	-
	**Conservative**	0	1	19	20	-
	**Total**	95	14	190	299	-

Notes: P value < 0.001.

*Abbreviations*: *ESRD* end-stage renal disease, *PD* peritoneal dialysis, *HHD* home hemodialysis, *HD* hemodialysis.

The independent associations with performing a home-based therapy are shown in Table 4. Compared to choosing facility-based HD, intending to perform a home-based therapy (PD or HHD) was strongly associated with initiating PD or HHD (adjusted OR 29.0 [95% CI 10.7-78.8] and 12.4 [95% CI 3.29-46.6], respectively; p < 0.001). The presence of CHF was a negative predictor of starting a home-based therapy (OR 0.25 [95% CI 0.11-0.58]; p < 0.001) whereas age, sex and DM were not. Factors which would be associated with more time to plan a home-based therapy, namely increased available time and a slower rate of loss of eGFR, were not independently associated with initiating a home-based dialysis therapy. Interestingly, a longer period of time from modality decision to initiation was associated with a decreased odds of starting a home therapy (OR 0.67 [95% CI 0.50-0.89]; p = 0.006).

**Table 4:** Multivariable logistic regression analysis of factors associated with home-based dialysis initiation and HD initiation using a catheter

**Outcome**	**Home-based RRT start**		**Start HD with a CVC**	
	**N = 299**		**N = 197**	
**Variable**	**Odds ratio [95% CI]**	**p-value**	**Odds ratio [95% CI]**	**p-value**
Intended modality^a^				
HD	Referent		Referent	
PD	29.0 (10.7, 78.8)	<0.001	3.73 (1.51, 9.21)	0.004
HHD	12.4 (3.29, 46.6)	<0.001	0.72 (0.23, 2.22)	0.56
Undecided/conservative	0.95 (0.17, 5.32)	0.96	4.58 (1.53, 13.7)	0.007
Age, per 10-years	0.92 (0.71, 1.18)	0.50	1.59 (1.15, 2.21)	0.006
Female sex	0.91 (0.48, 1.73)	0.78	3.24 (1.46, 7.20)	0.004
Diabetes mellitus	0.99 (0.51, 1.91)	0.97	1.57 (0.73, 3.39)	0.25
Congestive heart failure	0.25 (0.11, 0.58)	<0.001	3.74 (1.56, 8.96)	0.003
Weight, per 10-kg	0.94 (0.80, 1.09)	0.40	1.10 (0.92, 1.32)	0.29
eGFR decrease, per 1-ml/min/1.73 m^2^/yr^b^	0.99 (0.92, 1.07)	0.88	1.09 (0.99, 1.21)	0.093
Time-decision-initiation, years	0.67 (0.50, 0.89)	0.006	0.70 (0.51, 0.95)	0.024

Notes: ^a^reference group is HD. ^b^change in eGFR multiplied by (-1).

*Abbreviations*: *RRT* renal replacement therapy, *CVC* central venous catheter, *SE* standard error, *PD* peritoneal dialysis, *HHD* home hemodialysis, *kg* kilogram, *ml* milliliter, *m* meters, *eGFR* estimated glomerular filtration rate based on 4-variable MDRD equation, *yr* year.

Of the 197 total patients that initiated on HD (facility-based or HHD), 128 (65.0%) did so with a CVC. Table 3 shows the multivariable associations with initiating HD using a CVC. Female sex and increased age were associated with a CVC as the initial vascular access. Intending to perform PD was also associated with an increased probability of using a CVC (OR 3.73 [95% CI 1.51-9.21]; p = 0.004). Similarly, being undecided or intending conservative therapy was associated with an increased chance of ultimately initiating HD with a CVC as the initial vascular access (OR 4.58 [95% CI 1.53-13.7]; p = 0.007). Having more time between modality decision and initiation of dialysis was associated with a decreased probability of starting HD with a CVC (OR 0.70 [95% CI 0.51-0.95; p = 0.024).

Reasons why patients did not initiate on PD are outlined in Table 5. In patients initially intending to perform PD, 36.5% did not perform PD because of an earlier than anticipated requirement for dialsyis and 36.5% changed their mind, almost exclusively because of a preference for a hospital-based therapy.

**Table 5:** Reasons provided for patients not performing PD who intended to initiate PD

**Reason patient did not start on PD**	**N (%)**
**Medical reasons**	**38 (60.3)**
Acute start	23 (36.5)
Abdominal surgeries	5 (7.9)
Hernia	2 (3.2)
Obesity	2 (3.2)
Cognitive impairment	1 (1.6)
Abdominal aortic aneurysm	2 (3.2)
Shunt	1 (1.6)
Stroke	1 (1.6)
Patient in nursing home	1 (1.6)
**Patient reasons**	**24 (38.1)**
Patient preference for hospital-based treatment	23 (36.5)
Lack of space in home	1 (1.6)
**Unknown**	**1 (1.6)**

## Discussion

We demonstrated that patients who intend a home-based dialysis therapy still frequently initiate facility-based HD and are at higher risk for starting HD with a CVC as their initial vascular access. These results underscore the importance of the initial consideration of home-based dialysis therapies but also the need to continually re-evaluate and reinforce modality choices to improve preparedness for HD.

Utilization of home-based dialysis, including PD and HHD, remains low in North America [5]. Reasons for this are varied, however lack of appropriate pre-ESRD modality education [21,22] and/or late referral to a nephrologist [11] have often been suggested as reasons for high rates of utilization of facility-based HD. In our study, all patients were followed for at least 120 days and they received standardized, pre-ESRD modality education by a trained nurse. Despite this, home-based dialysis was the first actual modality in a minority of patients with many abandoning an initial preference for a home-based therapy. This suggests factors other than availability of pre-ESRD modality education and timing of referral influence the frequency with which patients utilize home-based dialysis.

Very few studies systematically evaluated the relationship between intended and first actual initiated dialysis modality and the potential negative consequences of a mismatch. A study by Liebman et al [23]. evaluated 217 patients who had received ESRD modality education at a single U.S. centre and went on to start dialysis. More than half of the patients selected PD as their initial choice modality but less than half of these ultimately initiated PD. A second study [24] found that an even higher proportion of patients than observed in the present study did not declare an intended therapy after modality education (49%). These studies were limited by either smaller sample size or incomplete data, however their broad similarity to the findings in our study are encouraging.

The reasons for abandoning the initial choice to perform a home-based therapy, particularly PD, are multiple. Amongst patients that intended to do PD but did not, more than 60% were for medical reasons. Although the majority of these were relative rather than absolute contraindications, they were perhaps the final contributor to the decision making process. Additionally, a preference change accounted for over one-third of those who did not initiate on PD as intended in this cohort. In our study we focused on patient’s medical characteristics to explain changes in preference. However, these characteristics explained very little of why patients who initially chose a home-based therapy did not start a home based therapy. More information on the psycho-social, environmental and logistical challenges that may contribute to why patients change their preference for home-based therapies is needed. A change in the patient’s perceived ability to perform PD between the time of initial modality decision and dialysis initiation may be partially responsible for the preference change and is consistent with the finding that an increase in this time interval was associated with reduced odds of starting a home-based therapy such as PD. Additionally, about one third of patients required dialysis earlier than first anticipated, leading to initiation of facility-based HD despite a declared intent to perform a home-based therapy. More careful planning of the timing of PD catheter insertion, the use of embedded PD catheters, or the availability of urgent PD catheter insertions may mitigate this issue.

Information on why a patient who initially selected HHD but started on a different modality was not available. The increased need for home resources associated with HHD (e.g. costs of home utilities and suitable water supply) may have played a role in the modality initiated. Further efforts are required to understand if the medical and non-medical events that alter patient preferences can be avoided and/or their effect on modality choice mitigated.

Changes in modality choice (i.e. patients initially undecided, wanted conservative care or wanted PD and ultimately started HD) resulted in a three to four-fold increase in the risk of initiating HD with a CVC. This risk could not be attributed to non-function of a PD catheter as such patients were counted as a PD start for the purposes of this analysis. Therefore, these results support the need for reinforcing and re-evaluating a patient’s modality decision over time. This suggests changes in decisions about dialysis modality late in the progression of CKD, particularly when unanticipated, may result in facility-based HD starts that may be suboptimal (e.g. initiating HD with a CVC rather than an AVF).

The finding that the presence of CHF was associated with decreased likelihood of starting a home-based therapy was somewhat unexpected. Previously published data indicating that the presence of CHF is associated with worse outcomes in PD patients as compared to HD [25] may have tempered nephrologists’ enthusiasm for PD in such patients, and directed this population towards HD. Alternatively, CHF may simply have been a marker of poor functional status and therefore less ability to ultimately perform a home-based therapy.

There are several limitations of this study. This is a single centre study which may limit generalizability to centres with different educational programs, patient mixes, and access to facility or home-based therapies. Despite this, the proportion of our patients that declared an initial intention to perform a home-based dialysis therapy and the proportion that actually initiated a home-based therapy are similar to those reported by other centres. Also, our study was retrospective with the inherent possibility to introduce bias in data collection. Much of the data, however, was collected prospectively and follow-up of patients was complete. As an observational study, our results are prone to residual confounding particularly as the determinants of initiating a home-based therapy are not certain from existing literature. For example, information regarding patients’ social supports and socioeconomic status may be relevant but was not available for our study [10].

## Conclusions

In conclusion, this study demonstrates there is a high probability that patients that intend to perform a home-based dialysis therapy, such as PD or HHD, are much less likely to initiate a home-based therapy compared to patients intending to perform facility-based HD. Additionally, these patients are disadvantaged by being more likely to start HD with a CVC rather than an AVF or arteriovenous graft. This study highlights the need for further research to better characterize time-dependent barriers to initiating home-based therapies and to develop interventions which will reduce these barriers. Patients who initially are appropriate for PD and prefer this modality, but have a change in their psychosocial or medical status that ultimately precludes performing PD at home, would be better served by earlier identification of this change leading to an improvement in vascular access planning.

## Abbreviations

AVF: Arteriovenous fistula; CAD: Coronary artery disease; CHF: Congestive heart failure; CI: Confidence interval; CKD: Chronic kidney disease; CVC: Central venous catheter; DM: Diabetes mellitus; eGFR: Estimated glomerular filtration rate; ESRD: End-stage renal disease; HD: Hemodialysis; HHD: Home hemodialysis; MDRD: Modification of diet in renal disease; PD: Peritoneal dialysis; RRT: Renal replacement therapy; SD: Standard deviation.

## Competing interests

Dr. Brimble has received an extramural grant from Baxter Corporation.

## Authors’ contributions

PTK carried out data acquisition and contributed to the study design. MW contributed to the design of the study, analysis plan and contributed critical revisions to the final manuscript. CMR contributed to the design of the study and data acquisition and contributed critical revisions to the final manuscript. KSB contributed to the design and coordination of the study, carried out data acquisition, performed the statistical analysis and drafted the manuscript. All authors read and approved the final manuscript.

## Pre-publication history

The pre-publication history for this paper can be accessed here:

http://www.biomedcentral.com/1471-2369/15/38/prepub
